# Performance and Accuracy of Lightweight and Low-Cost GPS Data Loggers According to Antenna Positions, Fix Intervals, Habitats and Animal Movements

**DOI:** 10.1371/journal.pone.0129271

**Published:** 2015-06-18

**Authors:** Marie-Amélie Forin-Wiart, Pauline Hubert, Pascal Sirguey, Marie-Lazarine Poulle

**Affiliations:** 1 Université de Reims Champagne-Ardenne, SFR Cap Santé, EA3800 PROTAL, Reims, France; 2 Université de Reims Champagne-Ardenne, CERFE, Boult-aux-Bois, France; 3 National School of Surveying, University of Otago, Dunedin, New Zealand; Institut Pluridisciplinaire Hubert Curien, FRANCE

## Abstract

Recently developed low-cost Global Positioning System (GPS) data loggers are promising tools for wildlife research because of their affordability for low-budget projects and ability to simultaneously track a greater number of individuals compared with expensive built-in wildlife GPS. However, the reliability of these devices must be carefully examined because they were not developed to track wildlife. This study aimed to assess the performance and accuracy of commercially available GPS data loggers for the first time using the same methods applied to test built-in wildlife GPS. The effects of antenna position, fix interval and habitat on the fix-success rate (FSR) and location error (LE) of CatLog data loggers were investigated in stationary tests, whereas the effects of animal movements on these errors were investigated in motion tests. The units operated well and presented consistent performance and accuracy over time in stationary tests, and the FSR was good for all antenna positions and fix intervals. However, the LE was affected by the GPS antenna and fix interval. Furthermore, completely or partially obstructed habitats reduced the FSR by up to 80% in households and increased the LE. Movement across habitats had no effect on the FSR, whereas forest habitat influenced the LE. Finally, the mean FSR (0.90 ± 0.26) and LE (15.4 ± 10.1 m) values from low-cost GPS data loggers were comparable to those of built-in wildlife GPS collars (71.6% of fixes with LE < 10 m for motion tests), thus confirming their suitability for use in wildlife studies.

## Introduction

Global positioning system (GPS) devices have improved the availability and accuracy of animal-relocation field studies and greatly enhanced wildlife research [1,2]. GPS provides large amounts of animal location data over short sampling intervals and large spatial scales, and the data are available day and night for various weather and terrain conditions [3,4,5]. Because of recent technological advances, GPS devices are now deployed to track large animals, such as the African elephant *Loxodonta africana* [6], fur seal *Callorhinus ursinus* [7] and white-tailed deer *Odocoileus virginianus* [8], and medium- and small-sized vertebrates, such as the European badger *Meles meles* [9], domestic cat *Felis catus* [10,11,12,13], common brushtail possum *Trichosurus vulpecula* [14], otter *Lutra lutra* [15], European hedgehog *Erinaceus europaeus* [16] and feral pigeon *Columbia livia* [17].

However, several constraints are associated with the use of built-in wildlife GPS receivers to monitor animals [18], particularly the financial costs of receivers (US $1000–2000 per unit), which limits their use and ability to provide conclusive inferences on population processes and behaviors [19,20]. However, to meet consumers increasing demand concerning location-aware technologies, GPS devices have now been adapted for everyday use, such as travel, sports or domestic pet tracking [21]. These lightweight, commercially available GPS tracking devices rely on similar hardware to those used for built-in wildlife tracking with the advantage of being more affordable (e.g., US $50 for data loggers, such as Catnip GPS CatLog or Mobile Action i-gotU GT-120, and US $300 for a data transmitter, such as Garmin Astro 320 Dog Tracking GPS). However, the performance and accuracy of these low-cost GPS data loggers must be carefully assessed because these devices were not specifically developed to track wildlife and may be inappropriate for such use or less reliable than built-in wildlife GPS.

The accuracy of large or lightweight GPS built-in wildlife tracking systems is generally considered to range from less than 5 m to 30 m [21,22], and positions can be determined repeatedly across a broader range of sampling intervals [3,5]. However, location failures over a pre-defined time schedule and/or abnormally high measurement errors of up to several kilometers can occur with contemporary GPS receivers [4,23,24]. A low fix-success rate (FSR) and high location errors (LEs) can lead to habitat misclassifications in studies on resource selection because differential rates of data loss among habitats could bias the identification of habitats and their importance in fulfilling life-history requirements [20,25]. These errors can also bias estimates of movement paths [26], and imprecise location information can influence estimates of home-range size and shape [27]. Assessing the effects of various factors on the performance and accuracy of wildlife GPS telemetry systems is essential for understanding the causes of GPS errors, which might be collar-brand dependent [28,29,30], and selecting the appropriate solution to correct errors and ensure that the GPS performance and accuracy meet the study's objectives [16,27]. Various solutions have been proposed for correcting bias produced by missing and/or imprecise locations in ecological analyses (review in [22], [31,32]). However, all of these solutions require assessments of the performance and accuracy of wildlife GPS under stationary tests [14,30,33,34] and controlled mobile tests [33,35,36] and/or to some extent by using returns from GPS devices mounted on free-ranging animals (i.e., data obtained by device retrieval or through remote transmission) [9,37,38].

Low-cost GPS data loggers have been deployed to study the post-release of hand-reared Irish hare *Lepus timidus hibernicus* [39], map sea behaviors of pelagic seabirds [40] and ranging behaviors of domestic cats *F*. *catus* in an urban environment [41], or quantify fine-scale movements of goats, sheep and dogs [42]. Two studies have evaluated GPS data logger spatial accuracy for tracking human movements by estimating point and line errors in an urban environment [43,44]. Another study used returns from GPS devices mounted on free-ranging Mountain brushtail possum *Trichosurus cunninghami* to consider GPS data logger performance [45]. However, the factors that can affect the performance and accuracy of low-cost and lightweight GPS data loggers for wildlife telemetry survey have not been assessed to date using stationary and motion controlled tests, and the same methods employed to test built-in wildlife GPS.

In stationary and motion controlled tests, GPS data logger errors are evaluated using indices that reflect the i) device performance in terms of the FSR according to the proportion of successful GPS scheduled attempts that resulted in successful location acquisition and ii) device accuracy in terms of the LE according to the Euclidean distance between GPS-estimated locations and “true positions”. Such errors are more likely to be obtained under specific environmental conditions and related to certain animal-related behaviors and technological factors that can spatially and temporally disturb signal transmission from satellites to receivers.

Typically, a greater number of satellite signals results in higher GPS receiver positioning performance and accuracy. However, the number of satellites available at any given time can be affected by physical obstructions between the receiver and satellites. The FSR and LE are largely influenced by environmental factors such as canopy closure [22,24,25,46] and topography [28,33,36], which can be approximated by sky availability and measures the quantity of visible sky from a given point. Furthermore, antenna positions < 45° from horizontal also affect the FSR [23,47] and LE [23,36] of GPS receivers, and technological factors such as the fix interval (i.e., frequency at which locations are collected) may also influence the FSR because of possible changes in satellite configurations over data acquisition intervals. Short fix intervals are generally associated with high FSR values without affecting the LE [28]. In addition, an increase in the fix interval affects the receiver's ability to determine locations in a timely manner (e.g., a 60-min interval requires a longer fix acquisition time than a 10-min interval) [36]. Furthermore, the duration of deployment may affect the FSR and LE values, although reference data on battery power are not presently available.

Aside from two studies that investigated lightweight built-in wildlife GPS collar performance and accuracy within urban [17] and suburban [14] environments, most of the research on GPS receivers has been performed in natural environments [8,25,34,38,48,49], which limits the ability to perform inferences on human-impacted landscapes used by medium-sized wild terrestrial species, such as the urban coyote *Canis latrans* [50], red fox *Vulpes vulpes* [51], raccoon *Procyon lotor* [52], ground squirrel *Spermophilus citellus* [53], and hedgehog *E*. *europaeus* [54].

The aim of this study was to investigate the performance and accuracy of lightweight and low-cost GPS data loggers in a rural environment to evaluate their suitability for wildlife research projects. Our objectives were therefore to 1) evaluate the consistency of same-brand commercially available GPS data loggers according to their duration of deployment; 2) characterize the influence of antenna position, fix interval and habitat on the rate of successful location acquisition and measurement accuracy using a stationary experimental arrangement; and 3) assess the effects on GPS performance and accuracy of motion across habitats by fitting medium-sized leashed mammals with GPS data loggers.

## Material and Methods

### Study area

This research was performed in a rural landscape in northeastern France. The study area was centered on two small villages (less than 200 inhabitants): Boult-aux-Bois (49°25’52”N, 4°50’33”E), which is characterized by a relatively flat topography (166–238 m), and Briquenay (49°24’19”N, 4°52’41”E), which is characterized by a more marked topography (130–258 m). The steepest terrain was located in a 3300-ha forested area (national forest of La Croix-aux-Bois) at the southeast border of the study area, whereas a low-relief landscape was found in the agricultural matrix composed of a mosaic of pastures, cultures, meadows, and groves (12%).

### GPS data logger

This study was performed with 40 commercially available GPS CatLog data loggers (22 g, Catnip Technologies Ltd, Hong Kong) suitable for tracking mammals weighing more than 800 g. These USB-rechargeable units consist of a GPS chipset (SiRF III), battery pack (380 mAh) and built-in patch antenna configured to search for satellites from < 38 sec in warm-start conditions (i.e., the receiver remembers its last calculated position but not the satellites that were in view because of changes in the satellite constellation) to < 60 sec in cold-start conditions (i.e., the receiver dumps all of the information because of fix intervals that are too long). The GPS data loggers function from -10°C to +50°C, and the units can store up to 64,000 fixes and can be programmed using an interactive interface with two different GPS sampling programs, including a continuous sampling interval from 1 sec to 60 min or scheduled sampling interval depending on the date and time. A successfully acquired location provides the date and time (Greenwich Mean Time, GMT) of the positional data, latitude, and longitude (based on the World Geodetic System 84). Additional information, including the altitude (m), speed (m/h), and distance from the last fix (m), is also recorded. When the GPS receiver cannot acquire a successful fix, the device does not record data. The data stored in GPS data loggers cannot be broadcast, and the unit must be recovered to retrieve the data via USB. The batteries of the 40 CatLog units were fully charged via USB before their deployment.

### Stationary unit tests

A stationary experimental arrangement of GPS units was designed to test the effects of antenna position and fix interval on the fix-success rate (FSR) and location error (LE). Twenty-four GPS data loggers were individually placed on the necks of 1.5-L plastic bottles (S1a Fig) and simultaneously deployed. The bottles were placed on small crossbars 15 cm above the ground and filled with a saline solution to mimic the ground plane of a medium-sized animal body [22]. The GPS bottles were spaced 1.5 m apart in a two-row by three-column grid network, with a grid cell containing four GPS bottles (S1b Fig). This test pattern was placed in an open habitat without topographical or vegetation obstructions to ensure favorable satellite views. Each GPS bottle was placed at a known benchmark with available geodesic coordinates.

The effects of the antenna at up and down positions (± 90° from horizontal) and three fix intervals (5-min interval, program A; 15-min interval, program B; and 1-h interval, program C) on the FSR and LE were simultaneously tested by deploying four GPS bottles for each configuration (S1b Fig). The GPS units remained at each benchmark for a minimum of 145 fix attempts to ensure that program A covered at least one full GPS satellite constellation cycle (approximately 12 h). The four GPS bottles with upward antennas and program B (S1b Fig) were also used to test the FSR and LE consistency of GPS data loggers over time. These four units recorded positions over 40 full cycles.

The influence of habitat on the FSR and LE was tested with 16 GPS bottles, which were simultaneously deployed with open habitat conditions. Four of the GPS bottles were placed in a dense stand closed conifer forest, four were placed in a barn, four were placed in a household, and four were placed just outside of a barn (1 m from a wall) (S1c Fig). Concrete walls and small sky views characterized the household habitat, whereas the barn presented a permeable corrugated roof. These GPS data loggers were deployed with fixed conditions (antenna up and program B) during two complete cycles (i.e., 97 expected fixes). Each GPS bottle was placed at known benchmarks in four habitats with available geodesic coordinates.

### Motion controlled tests

**Fig 1:**
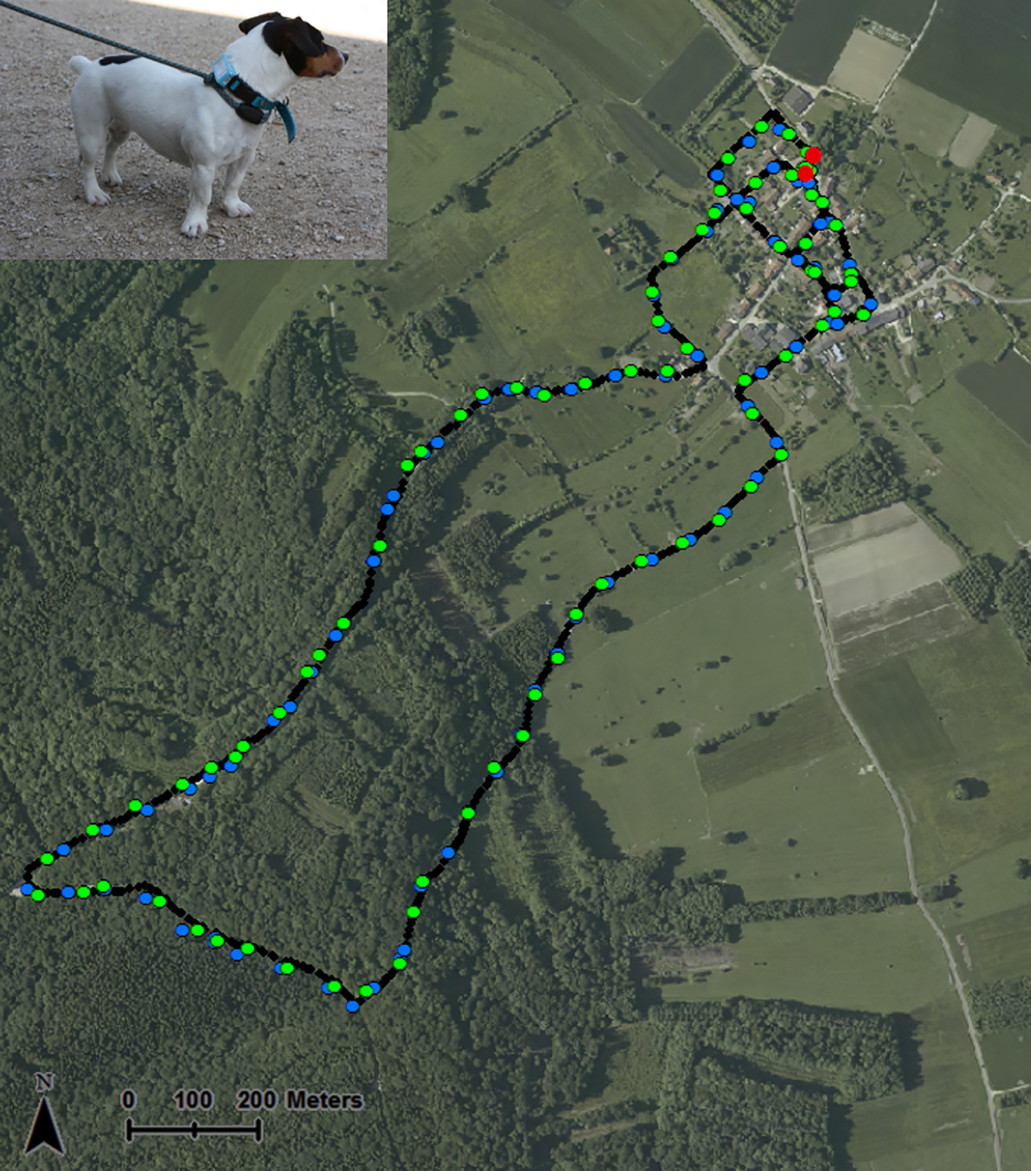
Motion controlled tests. Locations recorded by the Garmin control device (small dark dot) and two CatLog data loggers (large green and blue dots) with a fix interval set to 1 min along the path centered on Briquenay. Red points represent starting and ending points.

Two small dogs (6 kg and 8 kg) each carried one GPS data logger (Fig 1). The receivers were fixed to their collars, and the fix interval of the units was set to one minute. A research participant walked the dogs on a leash along the village streets and forest, meadow and edge paths. Two different routes (path one and two) were walked in similar weather conditions (clear skies and relative humidity of approximately 50%) in and around both villages leading to four trips (trip 1 = path 1, 1^st^ walk, trip 2 = path 2, 1^st^ walk, trip 3 = path 2, 2^nd^ walk, and trip 4 = path 1, 2^nd^ walk). The length of paths varied from 5.2 to 9.6 km with walking durations of 85 to 153 min. The reference paths were simultaneously recorded using a GPS Garmin Map 62s (root mean square (RMS) accuracy = ± 3–5 m) that collected positions at intervals of approximately 7 sec (range = 1–22 sec). This control device was held 1.5 m above the ground by the participant and remained at a constant distance from the dog equipped with the GPS data logger. Trajectories from the Garmin GPS control device were rediscretized in time to the second using linear interpolation to calculate the LE as the Euclidian distance between a CatLog-measured location and Garmin control location collected at the same time (Fig 1).

### Variables

Variations in the accuracy of the position estimates among collars were assessed by the horizontal and vertical errors of the 40 units deployed during the stationary unit tests. The FSR was obtained by dividing the number of successfully acquired locations by the number of scheduled attempts. The mean time of the fix acquisition (μTAF) was calculated by grouping units from each test configuration to understand the GPS performance. The LE was calculated for each positional fix as the Euclidean distance between each of the GPS-measured locations and corresponding ‘true’ reference positions defined as (i) the geodesic coordinates obtained from the Institut National de l’information Géographique et forestière (IGN) or (ii) fixes from a GPS Garmin Map 62s. The LE was determined as follows:

$$\text{LE}={\left[\Delta x²+\Delta y²\right]}^{0.5},$$

where Δ*x* and Δ*y* are the differences between the GPS-measured location and x and y reference positions, respectively.

The RMS of the LE (LE_RMS_), which measured the average location error of a GPS device extracted from a population of *n* positional fixes, was also calculated for each test configuration and habitat during stationary tests and for each trip and habitat during the motion tests. This measure assists in the selection of buffer sizes around GPS locations when performing habitat-related spatial analyses on free-ranging animals [22]. The LE_RMS_ was computed as follows:

$${\text{LE}}_{\text{RMS}}={\left[\frac{{\text{LE}}_{1}²+{\text{LE}}_{2}²+\ldots +{\text{LE}}_{n}²}{n}\right]}^{0.5}.$$

The arithmetic mean (μLE) and median (*m*LE) of the location error of *n* fixes were also calculated for comparisons with previous studies for each test configuration and habitat during the stationary tests and for each trip and habitat during the motion tests. The interquartile range (LE_IQR_) was computed as an additional measure of LE dispersion (i.e., the precision of each configuration) for each test configuration in open habitat. The outlier LE values that occurred with all of the GPS devices [22] were identified as data that do not fall within three standard deviations of the mean LE value. The number of outlier values and LE_RMS_ values without outliers were also computed for each collar deployed during the stationary unit tests and for each habitat traversed during the motion tests.

Sky availability (*V*_*d*_) was also computed as a topographic factor. *V*_*d*_ measures the quantity of visible sky from a given point and is computed based on a 25-m digital elevation model (DEM) by determining the horizon angle *H(φ)* in 64 discrete azimuth angles *φ*. *V*_*d*_ is then defined as the ratio between the solid angle subtended by the horizon lines and that of the entire upper hemisphere (Ω = 2π, visibility = 1, [55]). Therefore, sky availability was computed as follows:

$$\begin{array}{c} V_{d}=\frac{1}{2\pi }\int_{0}^{2\pi }\int_{0}^{H(\varphi )}\text{sin}\theta d\theta d\varphi \\ =\frac{1}{2\pi }\int_{0}^{2\pi }\left[1-\text{cos}H(\varphi )\right]d\varphi \end{array}$$

This measure can be considered a factor for the quality of the fix by quantifying the probability of the presence of a sufficient number of satellites under an appropriate geometry. *V*_*d*_ ranges from 0 for totally obscured sky conditions, such as the bottom of an incised valley, to 1 for an unobstructed horizontal surface. To exclusively assess the effect of the behavioral, technical, and environmental factors on GPS performance and accuracy, the units were deployed at locations that exhibited high sky availability (0.87–1.00) during the stationary unit tests [33].

### Data analysis

The coordinates were projected from both the GPS CatLog devices and Garmin Map 62s to Universal Transverse Mercator (UTM) for analysis. The geographical analyses were performed using ArcGIS 10 (ESRI, Redlands, CA, USA). The model selection approach was based on the Information Theoretic Approach and performed with the Akaike Information Criterion (AIC) following the protocol described by Zuur et al. [56]. The best model was validated by graphic inspection [56,57]. All of the statistical analyses and modeling were performed in the statistical software program R 3.1 [58], and a value of alpha = 0.05 was used for all tests.

The consistency of GPS data-logger performances was initially assessed by the FSR differences between the collars from the upward antenna and program B test configuration. Significant differences between those units (fUnit) (Table 1) relative to the log-transformed LE values from two full cycles (i.e., 97 possible fixes) were then tested using a one-way analysis of variance (ANOVA). Finally, the proportion of outliers (Nout) and median of the LE values (*m*LE) from the antenna-up receivers and program B logging over 40 full cycles (fTime, Table 1) were tested using linear and non-linear regressions to determine the consistency of the GPS data loggers over time and assess the potential effect of battery power.

**Table 1:** Variables used in the evaluation of GPS performance and accuracy during stationary and controlled motion tests.

**Explanatory variables**	**Type**	**Description**
fTime	Continuous	Number of the 12-hours cycle (12–480 hours)
fAntenna	Categorical	Antenna positions: (1) upward; (2) downward
fFix	Categorical	Fix intervals: (A) 5-min; (B) 15-min; (C) 1-h
fHabitat	Categorical	Habitat types: (1) open; (2) barn; (3) forest; (4) household; (5) wall
fUnit	Categorical	Number code identifying GPS unit individually
fHabCross	Categorical	Habitat crossed: (1) open; (2) edge; (3) forest; (4) village
*Vd*	Continuous	Sky availability (0–1)
fDay	Categorical	Date of the controlled motion test
**Response variables**	**Type**	**Description**
Nout	Count	Proportion of outlier values
mLE	Continuous	Median LE value of a given cycle
FSR	Binary	Scheduled attempts resulting in successful (1) or unsuccessful location (0)
LE	Continuous	Measurement error of GPS locations relative to their “true” value (m)

**Fig 2:**
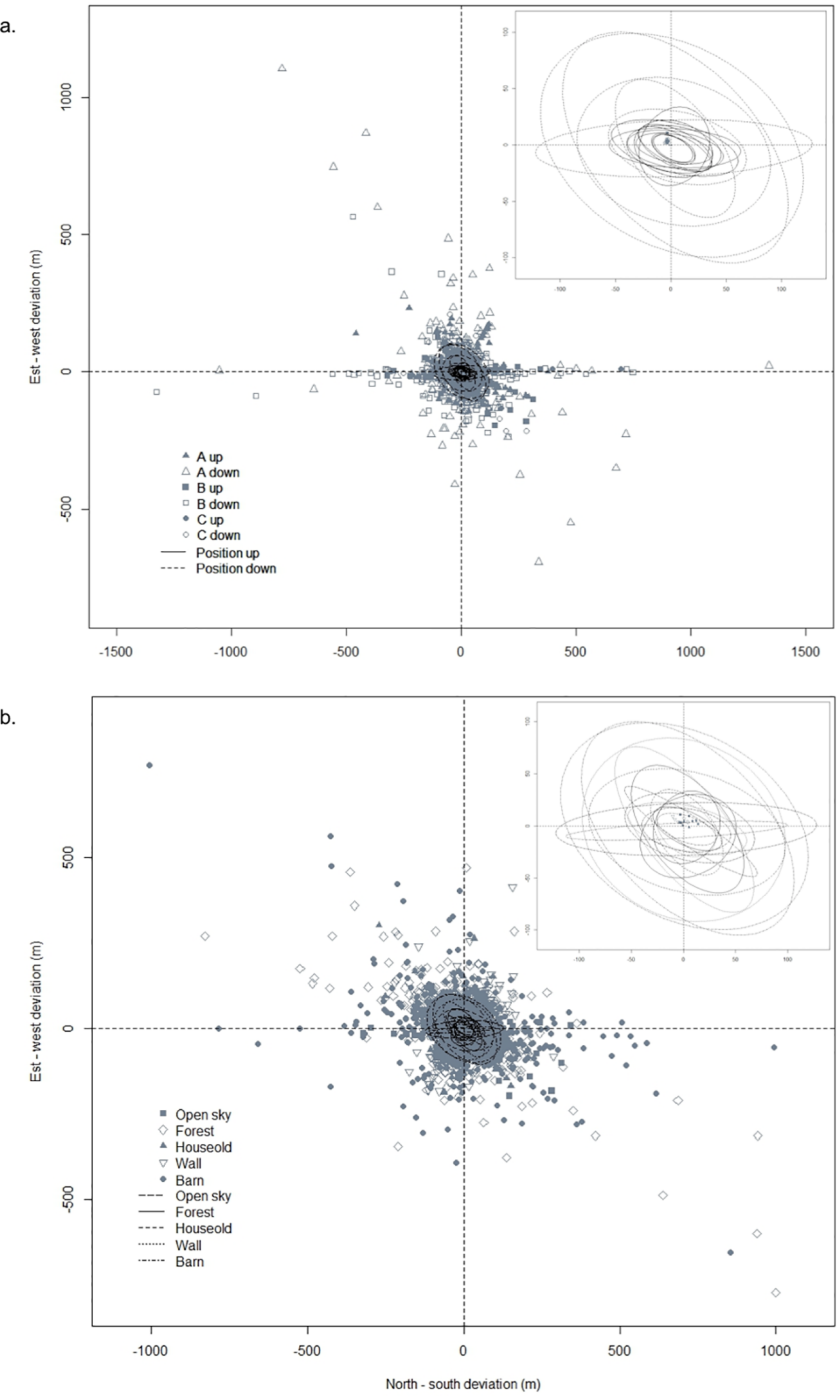
Systematic error. Location errors from 39 GPS CatLog data loggers relative to the National Geodetic Survey coordinates obtained of (a) 700–2300 fixes per unit under antenna positions (up or down) and fix intervals (program A, program B or program C) in an open habitat and (b) 250–2300 fixes per unit under different habitats with fixed conditions. Ellipses represent 95% confidence areas and centroids represent the average location error.

The tendency of the estimated position accuracy to fluctuate among GPS data loggers according to the configuration (Fig 2a) and habitat (Fig 2b) during the stationary unit tests was investigated by descriptive statistics (Fig 2). The FSR was computed for each unit involved in the stationary unit tests (Table 2) and for each track and habitat crossed during controlled motion tests (Tables 3 and 4). For each stationary unit test configuration and successful location fix, we calculated the LE, LE_RMS_ with and without outlier values, and proportion of fixes with LE < 10 m (Table 2). These variables were also calculated for each trip walked and habitat crossed during the controlled motion tests (Tables 3 and 4).

**Table 2:** Results from stationary unit tests performed with 40 low-cost CatLog GPS data loggers: the fix success rate (FSR) ± standard deviation (SD), mean time of the fix acquisition (μFAT), root mean square of the location errors (LE_RMS_), mean location error (μLE), median location error (*m*LE), percentage of fixes with LE < 10 m, the mean number of outliers per unit (N outliers) and root mean square of the location errors after the removal of outliers (LE_RMS_ without outliers) for positional fixes collected from for two antenna positions, three fix intervals programs and four habitat types.

Habitat	Fix interval	Antenna position	FSR±SD	μFAT	LE_RMS_ (m)	μLE (m)	*m*LE (m)	Percentage LE < 10 m	N outliers	LE_RMS_ without outliers (m)
Open	Program A	Up	1.00 ± 0.02	4 min 39 sec	12.7	4.1 ± 4.1	3.2	92.1 ± 5.1	3.3 ± 0.5	5.8
	(5 min)	Down	1.00 ± 0.05	5 min 02 sec	65.4	13.9 ± 14.2	9.6	50.3 ± 10.5	4.3 ± 1.0	19.9
	Program B	Up	1.01 ± 0.08	13 min 43 sec	12.4	4.0 ± 4.4	2.7	90.4 ± 2.8	3.3 ± 1.0	5.9
	(15 min)	Down	1.01± 0.09	12 min 56 sec	21.0	8.7 ± 8.8	6.2	72.4 ± 5.5	3.0 ± 1.4	12.3
	Program C	Up	1.01 ± 0.10	57 min 04 sec	10.3	4.3 ± 4.4	3.0	91.9 ± 4.4	2.0 ± 0.8	6.1
	(1 h)	Down	1.01 ± 0.12	54 min 47 sec	20.0	8.7 ± 8.2	6.3	71.4 ± 6.2	3.0 ± 0.8	11.9
Household	Program B	Up	0.20 ± 0.17	38 min 14 sec	47.6	31.7 ± 32.3	18.3	13.8 ± 9.9	0.3 ± 0.6	45.2
Barn	Program B	Up	0.98 ± 0.11	14 min 09 sec	65.0	31.1 ± 30.7	21.0	13.3 ± 3.8	2.3 ± 0.5	43.7
Proximity to building	Program B	Up	0.75 ± 0.27	20 min 38 sec	31.3	20.4 ± 15.4	16.9	24.2 ± 10.7	1.8 ± 0.5	25.6
Closed conifer forest	Program B	Up	1.07 ± 0.03	12 min 51 sec	68.9	26.8 ± 31.0	17.4	26.3 ± 6.5	2.5 ± 0.6	40.9
**Total**			0.90 ± 0.26	-	35.5	15.4 ± 10.1	10.5	54.6 ± 31.3	2.6 ± 1.0	21.7

**Table 3:** Results per trip from motion controlled tests performed on two low-cost CatLog GPS data loggers mounted on two dogs: the number of fixes acquired (N), distance walked (m), walking time (min), fix-success rate (FSR), percentage of fixes with LE < 10 m, root mean square of location errors (LE_RMS_), mean location error (μLE) ± standard deviation (SD), median location error (mLE), number of outliers per trip (N outliers) and root mean square of the location errors after the removal of outliers (LE_RMS_ without outliers).

Trip	N	Distance (m)	Time (min)	FSR (%)	% LE < 10 m (%)	LE_RMS_ (m)			Outliers	
							μLE (m)	mLE	N outliers	LE_RMS_ without outliers (m)
1	172	9 662	153	112.4	72	13.3	8.7 ± 6.0	7.4	2	10.5
2	91	5 224	85	107.1	66	24.9	10.1 ± 10.8	7.5	3	14.7
3	92	5 224	95	96.8	74	22.4	8.1 ± 7.1	6.4	4	10.8
4	128	9 638	124	103.2	63	13.2	9.4 ± 5.9	8.3	2	11.1
Total	483	29 748	457	104.9 ± 6.6	69 ± 5	18.5 ± 6.1	-	-	2.8 ± 1.0	11.8 ± 2.0

**Table 4:** Results per habitat from motion controlled tests performed on two low-cost CatLog GPS data loggers mounted on two dogs: the number of fixes (N), mean location error (μLE) ± standard deviation (SD), median location error (mLE), range of location errors, percentage of fixes with LE < 10 m and root mean square of location errors (LE_RMS_).

Habitats	N	μLE (m)	mLE (m)	Range of LE (m)	LE < 10 m (%)	LE_RMS_ (m)
Edge	77	7.7 ± 5.2	6.4	[0.2–24.8]	76.6	9.2
Forest	165	13.3 ± 10.2	10.8	[0.5–114.4]	44.2	16.8
Open	98	6.8 ± 3.6	6.6	[0.6–17.6]	79.6	7.6
Village	143	6.8 ± 3.9	6.5	[0.3–18.7]	86.0	7.8

When testing antenna positions and fix intervals, all of the measurements were calculated for one complete cycle for program A, three cycles for program B and 12 cycles for program C to attempt 145 fixes. When testing the influence of habitat types, all of the measurements were calculated for two cycles to attempt a minimum of 97 fixes. To manage variations in the logging rates, the FSR accounts for the actual maximum number of fix attempts rather than the anticipated value for each test configuration.

The effects of antenna position (fAntenna) and fix interval (fFix) (Table 1) on the FSR were initially assessed between each test configuration. The influence of the same explanatory variables and interaction between fAntenna and fFix on LE was then modeled through a linear mixed-effects model (LMM, *nlme package*). The response variable included all of the log-transformed LE values of the fixes for the 24 benchmarks and up to 145 fixes per unit. The variable GPS (fUnit, Table 1) was used as a random term to consider non-independence among the fixes collected by each GPS receiver [14,33]. Thus, five possible models (alternative hypotheses and a constant null model) were compiled (S1 Table). Finally, the effects of the same explanatory variables on the log-transformed LE_IQR_ values were tested using a two-way ANOVA with permutation tests (*lmPerm package*), and post-hoc tests were performed for pairwise comparisons.

The probability of a fix attempt succeeding in a habitat type (fHabitat, Table 1) was modeled using a fixed-effect logistic regression. The fits of the full and reduced models were compared using a deviance test (*G*²) as an alternative to an individual parameter-based approach. Odds ratios with 95% confidence intervals were calculated for significant levels of the categorical predictor variable. Moreover, the influence of the same explanatory variable on the log-transformed LE values using a nested ANOVA (*lme* procedure) was investigated. GPS data loggers used within each habitat type (fUnit, Table 1) were treated as random terms.

The effects of habitat crossed (fHabCross), *V*_*d*_, day of the path walked (fDay) and receiver (fUnit) (Table 1) on the LE were modeled through a Gaussian generalized-linear model (GLM). Following the method of Zuur et al. [59], data were screened using a multiple correspondence analysis (MCA, *ade4* package) prior modeling to avoid correlated explanatory variables within the identical model. The response variable included all of the log-transformed LE values of the fixes from each GPS unit. Because the *V*_*d*_ values ranged between 0.84 and 1.00, the explanatory variable was centered on zero in all models. Eleven possible alternative—hypothesis models and a constant null model were considered (S2 Table).

### Ethics statement

Stationary unit tests were conducted with permission from private landowners and National Forestry Office by placing a stationary experimental arrangement of GPS units on private properties and in the national forest of La Croix-aux-Bois.

Motion tests were performed with two small dogs carrying GPS units on their collars to imitate the size and movement of a medium-sized terrestrial mammal and record the potential GPS unit antenna movement, variable speed and trajectory. In addition to collaring the dogs with GPS units in accordance with the manufacturer’s directions, the dogs were not handled or transported in an unfamiliar environment, and they did not perform unusual activities because they were used to wearing a collar and walking on a leash in various habitats. The tests were conducted under the supervision of an experienced behavioral biologist with the permission and in the presence of the dog owners, and they complied with the International Guiding Principles for Biomedical Research Involving Animals guidelines. Additionally, the municipalities of Boult-aux-Bois and Briquenay and National Forestry Office approved the paths into the villages and surrounding environments.

## Results

### Stationary unit tests

#### GPS consistency

The four units located in the upward antenna and program B test configuration (S1b Fig) had an FSR > 1. This result indicates that all of the expected positional fixes and additional fixes were obtained in open habitat because GPS data loggers presented a variation in logging rate (e.g., μFAT of 13 min 43 sec compared with the expected 15 min, Table 2). Similar variations in the logging rate against the fix interval scheduled (i.e., a μFAT lower than the programmed fix interval for the cycle) have previously been observed with commercially available GPS data loggers deployed during stationary tests [45].

After re-calculating the FSR to account for the actual maximum number of fixes attempts rather than the anticipated number (i.e., 106 versus 97 scheduled attempts), the FSR values remained high (FSR = 1.01 ± 0.08). The LE values between the units tended to differ significantly when simultaneously deployed (F_3,420_ = 2.28, p = 0.08), with LE values ranging between 0.2 and 163.2 m (median = 3.0 m). The frequency of outlier values was not linearly related to the number of full cycles the units were deployed (R^2^ = 0.02, p = 0.42). The median of the LE values also was not significantly explained by a cubic regression (R^2^ = 0.56, p = 0.89, S2 Fig).

#### Systematic error

The GPS data loggers provided coordinate errors of 3.3 m east and 3.6 m north on average compared with the 24 national geodetic survey (NGS) benchmarks involved in the stationary deployment in various configurations (Fig 2a). One of the GPS data loggers produced a mean location north of all other units, and the precision of the position estimates varied among the GPS units. In different habitats, the GPS data loggers provided coordinates that showed similar biases (2.9 m north and 5.1 m east in open habitat, 4.9 m north and 2.5 m east in forest, 5.5 m north and 6.2 m west near buildings, and 9.9 m north and 5.3 m east in barn habitat; Fig 2b). An exception was in the household habitat, which presented coordinates that showed north-south and east-west deviations depending on the units. Overall, the biases varied among the GPS units within each habitat.

#### Effect of antenna position and fix interval

FSR values > 1 were obtained for all of the tested configurations once the actual maximum number of fix attempts (rather than the anticipated number of 145 possible fixes) was accounted for in the calculations (Table 2). The LE_RMS_ values ranged from 10.3 m to 65.4 m and increased when the unit’s antenna was down and over short fix intervals (Table 2). Filtering outliers from each unit improved the LE_RMS_ values ranging from 5.8 m to 19.9 m (Table 2) depending on tested configurations. Thus, the size of the dispersion buffers significantly decreased when filtering the outliers. The relative share of fixes with LE < 10 m was higher for each program when the antenna was up (> 90%, Table 2) and lower when the antenna was down, particularly for program A (Table 2). This result indicates that > 90% of the locations acquired can be accurate enough for fine-scale analyses in open habitats when the GPS data logger antenna is up.

The likelihood ratio test applied to the model selected for LE indicated that the model with random effects was significantly better than the model without random effects (*L* = 28.22, *df* = 1, p < 0.001). The top-ranked model included fAntenna and fFix, and the interaction between these two variables (R^2^ = 0.21, S1 Table). The antenna position had the strongest effect on LE, with LE values that were—1.14 ± 0.10 SE lower (on the logarithm-transformed scale) when the GPS unit’s antenna was up than when the antenna was directed downward (p < 0.001). The fix interval also influenced the LE, with lower values obtained for programs B and C compared with that of program A (coefficient = -0.44, SE = 0.10, p < 0.001 and coefficient = -0.39, SE = 0.10, p < 0.001 for programs B and C, respectively). Regardless of the antenna position, the LE values obtained with programs B and C were lower than those obtained with program A, and this difference persisted when the GPS receiver’s antenna was up (coefficient = 0.35, SE = 0.14, p < 0.05 and coefficient = 0.39, SE = 0.14, p < 0.05 for antenna up and program B, and antenna up and program C, respectively). Therefore, once a position was acquired, its accuracy was higher when the antenna was directed upward with fix intervals of 15 min and 1 h than with a fix interval of 5 min.

A two-factor ANOVA using permutation tests showed that antenna position had the strongest effect on the LE_IQR_ values (F_1,20_ = 5.9, p < 0.001), with LE_IQR_ values increasing (i.e., increased dispersion of locations caused decreased precision) when the GPS unit’s antenna was down. The fix interval also influenced the LE_IQR_ values (F_2,20_ = 0.4, p < 0.05), with program A increasing the dispersion of positional fixes (p < 0.05) compared with that of program C, whereas program B only presented a tendency to increase the dispersion of positional fixes (p = 0.07) compared with that of program C.

#### Effect of habitats

The LE_RMS_ values between habitats ranged from 12.4 to 68.9 m (Table 2). When filtering outliers, the highest LE_RMS_ value was observed in the household habitat (Table 2). Following outlier removal, LE_RMS_ values remained relatively high compared with that of LE filtering performed for the GPS units deployed in an open habitat except for that of units deployed near buildings (Table 2). Moreover, fixes with LE < 10 m were scarce for all tested habitats compared with the open habitat condition (Table 2).

The FSRs presented significant differences according to fHabitat (*G*² = 1183.3, *df* = 4, p < 0.001, R^2^ = 0.52). The odds of a fix attempt succeeding for units located in the barn were equivalent to those for units located in the forest and open habitats (Table 2). However, the odds of obtaining successful locations when the units were in a household and at proximity from a wall were 72 and 6 times lower (household: FSR = 0.20, OR = 0.01, 95% IC: 0.01–0.02; proximity from a wall: FSR = 0.75, OR = 0.17, 95% IC: 0.11–0.27), respectively, than those for units located in a barn (Table 2). In the household habitat, three of the four units successfully logged at least one location (i.e., one collar never collected positions).

A likelihood ratio test applied to the model selected for LE indicated that the model with the random effects was significantly better than the model without random effects (*L* = 30.43, *df* = 1, p < 0.001, R^2^ = 0.47). The LE values for the GPS units located in open habitat and at proximity from a wall were -2.09 ± 0.15 SE (p < 0.001) and -0.36 ± 0.16 SE lower (p < 0.05), respectively, than the LE values computed with GPS units deployed in a barn (on the logarithm-transformed scale). LE values produced by units located in the forest habitat and households did not differ from those produced from GPS located in a barn. Therefore, the GPS data loggers located in an open habitat and at proximity from a wall produced more accurate positional fixes than units located in human settlements (households and barns) and in forest habitat.

### Motion tests

The trips yielded high FSR values that ranged from 0.97 to 1.12 with μFAT values < 1 min (Table 3). The LE_RMS_ values ranged from 13.2 m to 24.9 m with outliers and from 10.5 to 14.7 m without outliers. The highest LE_RMS_ values without outliers corresponded to trip 2 and trip 3 performed in and around Briquenay village, which is characterized by a more marked topography than Boult-aux-Bois. Outlier values occurred for each trip and were exclusively located in the forest. Removing these outliers reduced the LE_RMS_ values, particularly for the shortest path (Table 3). The highest LE_RMS_ value according to habitat was obtained for the forest habitat (16.8 m, associated with a large range of LE values) and then the edge habitat, whereas the village and open habitat had similar LE_RMS_ values (Table 4). An average of 69 ± 5% recorded fixes had an LE < 10 m. The proportion of fixes with an LE < 10 m was higher in the edge, open and village habitats compared with the forest habitat (Table 4).

The top-ranked model only included fHabCross (R^2^ = 0.17, S2 Table). During motion tests, GPS CatLog appeared to produce LE values that were 0.68 ± 0.11 SE higher (p < 0.001) when the fixes were acquired in the forest habitat than when the fixes were acquired in the edge habitat (on a log-transformed scale). The LE values were equivalent when the fixes were acquired in the open habitat, village and edge habitats. Therefore, the LE values significantly increased with increasing canopy cover.

## Discussion

This is the first study to investigate the performance and accuracy of low-cost GPS data loggers using the same testing methods employed to test expensive built-in wildlife GPS devices. The CatLog data logger fix-success rate (FSR) appears to be consistent among units, whereas location errors (LEs) have a tendency to vary among units. A similar finding was reported in a recent study that performed stationary tests on lightweight commercially available Sirtrack collars [48]. Thus, testing individual GPS data loggers before deployment on animals is recommended. The GPS data loggers tested here were also temporally reliable during the entire battery lifespan. A recent study successfully increased the GPS data logger lifespan by increasing the battery pack [45]. Our findings suggest that similar modifications could be performed on CatLog data loggers without affecting their performance and accuracy when tracking animals over a longer period.

During the stationary tests, the GPS units provided coordinates that deviated by 4 m on average in both the east-west and north-south directions relative to the NGS benchmarks. These results fell within the ranges of results recently reported for commercially available built-in wildlife GPS collars (e.g., Lotek large GPS collars with 4 m west and 10 m south mean deviations [29] and lightweight (105-g) Sirtrack collars with 5.4 m east and 6.0 m north mean deviations [48]). These systematic error ranges may not be detrimental depending on the study objectives (e.g., they must be considered when studying the fine-scale selection of habitat patches or redrawing precise animal paths) and animals surveyed (e.g., dimensions of habitat patches used by animals and their movement patterns).

All but one commercially available GPS data logger deployed during the stationary unit tests operated normally. The single unit that failed to fix positions in the household habitat did not undergo a technical failure and was found to function properly; thus, we believe that it did not receive sufficient satellite signals to compute the fixes. Based on all of the antenna positions and fix intervals, the average stationary FSR obtained for low-cost GPS data loggers was relatively high compared with the results reported in similar studies that have performed stationary tests with larger GPS collars (e.g., ATS collars, 1.00–0.76 [23]; G2000 ATS collars, 0–1.00 [47]; 3580 Telonics collars, 0.92–1.00 [28]). The results of the stationary tests did not identify an antenna oriented at < 45° from horizontal or different fix intervals as potential sources of FSR variation, which is inconsistent with previously reported results in the literature (Table 5). Thus, throughout this study, the movements of the CatLog GPS data loggers that were deployed on free-ranging animals and mimicked here by the antenna positions had no effect on the GPS data logger performance.

**Table 5:** Summary of GPS FSR and LE values reported for stationary and motion controlled tests during this study and for built-in wildlife GPS collars under various test conditions. The reported values are the range of mean values reported across the studies indicated.

Tests	Variables	Effect on		GPS device
		FSR ^a^	LE (m) ^b^	
**Stationary**	**Collar orientation**			
	135–180° from vertical	No effect	5–10	CatLog data logger [This study]
		0.93,12–24	17	ATS collars [23]
		0.74,44–99	-	G2000 model (ATS) [47]
		-	17	3300 L (Lotek) [36]
	**Fix interval**			
	5-min	No effect	13.9 *	CatLog data logger [This study]
	15-min		8.7 *	
	1-h			
	30-min to 6+h	0.96, 1–8	No effect	3580 model (Telonics) [28]
	10-min and 1-h	-	No effect	3300 L (Lotek) [36]
	**Habitat**			
	Open sky	No effect	4	CatLog data logger [This study]
	Proximity to building	0.82, 20	20	
	Household	0.22, 80	32	
	Barn	No effect	31	
	Close conifer forest		27	
		0.02	-	105-g Sirtrack [33]
	Native forest	0.49–0.63		
	Canopy cover > 70%	0.02–0.37	19–30	Built-in wildlife GPS [22,34,36]
**Motion**	**Motion technique**			
	Leashed dogs	1.05	11.8^(^ ^†^ ^)^, 69^(^ ^‡^ ^)^	CatLog data logger [This study]
	Sled-like device	0.90	14.1–50.2^(^ ^†^ ^)^	105-g Sirtrack [33]
	Attached devices (car)	0.87 (forest)	-	3300 L (Lotek) [36]
	Animal returns	1.00 ± 0.24	70^(^ ^‡^ ^)^	I-gotU GT-120 data logger [45]
	Humans (open habitat)	-	10.3^(^ ^†^ ^)^	I-gotU GT-100 data logger [43]
	Humans (village)	-	< 7	I-gotU GT-100 data logger [44]

^a^ Mean FSR values are reported and possibly followed by a reduction rate (%).

^b^ Mean LE values are reported for the stationary tests, and LE_RMS_

(^†^) and the proportion of fixes with LE < 10 m

(^‡^) are also reported for the motion tests.

* Mean LE values considering the interaction between the variables fAntenna (downward) and fFix.

Moreover, except for the household habitat and proximity to buildings, the FSR values from the units deployed in the different habitats were comparable to those reported by the commercially available Lotek 3300 L GPS collars [24] and were larger than those reported by stationary studies performed with built-in lightweight GPS collars (e.g., 0.91 in a suburban environment [14]; 0.92 in New Zealand farmland habitat [48] and 0.89 in a natural habitat [11]) or with larger collars (0.96 [28]). Thus, the performance of low-cost GPS data loggers appears to be as good as commercially built-in GPS collars.

Modeling the FSR according to habitat emphasized that complete or partial sky obstruction influenced data acquisition because the FSR decreased as the amount of open sky decreased. For example, positioning the GPS data logger in a household can result in an 80% reduction in the FSR, whereas positioning it near buildings can result in a 25% reduction because of poor satellite view. These results confirm those of Adams et al. [14], who showed that particular suburban habitat types influenced the FSR because of variations in vegetation complexity and distance to buildings. Although all of the benchmarks were in locations with high sky availability, the GPS units in obstructed environments behaved identically to those deployed in steep terrain [28]. In our study, the FSR was not dependent on canopy cover, which is inconsistent with the results of numerous studies (review in [22]). However, a recent study also reported that the FSR was not related to canopy cover (0.98–1.00 [24]). This difference may have occurred because the coniferous forest where the GPS units were deployed was not as closed-in as native forests [33] (Table 5).

Filtering out the outlier values led to a decrease in LE_RMS_ values from only 2 m to up to 45 m. Such variations may be associated with the number of satellites available to the receiver at a given time because the decrease in LE_RMS_ values followed a trend from non-obstructed habitats (open habitat) to partially obstructed (proximity to buildings) and obstructed habitats (forest, barn and household areas). Similar results were observed by Recio et al. [33], who located GPS collars under mature pine and native forests. The number of outliers that were documented in our stationary tests was similar to that in previously reported results using built-in wildlife GPS collars. In this study, 13 locations had errors of > 300 m (three fixes with downward antennas and 10 fixes in various obstructed habitats) out of 5046 successfully acquired locations. Other studies presented results that contained 2 outliers with errors > 300 m out of 6359 locations obtained with ATS collars [23] and 27 outliers with errors > 300 m out of 3441 locations obtained with Lotek 3300 L GPS collars [24]. Moreover, the range of the average and/or median LE values for the positional fixes obtained during the stationary tests were comparable to those reported by studies performed in natural environments [28,33,36] and an urban environment (μLE = 30.1 m) [14] with built-in wildlife GPS collars.

Our LE_RMS_ values were larger than those obtained with similar commercially available GPS data loggers (LE_RMS_ = 4.4 m) under open sky conditions [43]. A comparison of the different fix intervals and test durations used in our stationary unit tests and in tests performed with built-in wildlife GPS collars (e.g., 15 min to 13 h [28]) with those achieved by Vazquez-Prokopec et al. [43] may help to explain the inconsistency in the results. Vazquez-Prokopec et al.'s study used a 2-sec interval for 2-min tests, thus allowing the GPS data loggers to achieve positions under ‘hot-start’ conditions, which perform fast positional fixes because the GPS device remembers its final calculated position and visible satellites attempt to collect new positional data; however, the fix intervals used in this study only achieved fixes under ‘warm-start’ (5 min and 15 min) or ‘cold-start’ (1 h) conditions. For these conditions, the GPS device might remember its last calculated position but not the satellites that were visible because of a change in the satellite constellation (i.e., warm start). Alternatively, the device might dump all the information because of an extended fix interval (i.e., cold start). Consequently, the changes in the satellite constellation [36] and start conditions [60] had considerable influence on the GPS devices’ performance and accuracy.

If the antenna position did not influence the FSR, then it was shown to greatly influence the GPS accuracy, which was supported by the LE_IQR_ values and proportion of fixes with LE < 10 m. These results were consistent with those from studies performed with built-in wildlife GPS collars that tested collar orientation [23,36] (Table 5). Our findings stressed that the position of the GPS data logger antenna had an important effect on GPS accuracy because it reduced the optimal antenna orientation related to specific behaviors of collared animals (i.e., scratching the GPS collar, climbing trees, and lying on side or resting).

In general, the shorter the fix interval, the higher the accuracy because the satellites are continuously tracked at short time lags (i.e., ‘warm-start’) [28,36]. Contrary to previous studies, our results indicate that the fix interval negatively influenced the measurement error, to a lesser extent when the antenna is directed upward (e.g., *m*LE = 3.2 m for 5 min against 2.7 m and 3.0 m for 15 min and 1 h, respectively), but markedly when associated with a collar orientation of—90° from the horizontal and a 5-min fix interval. Given our findings, one possible explanation is that short fix intervals might imply more temporally autocorrelated positional fixes (i.e., the accuracy of the fix acquired at time *t + 1* depends on the accuracy of the fix acquired at time *t* by the GPS data logger). Therefore, when the number of satellites available to acquire a positional fix is low or when the GPS signal is reflected on the ground, the measurement error increased (e.g., 50.3% of fixes with LE < 10 m). However, longer fix intervals with positional fixes that were less correlated (15 min) or not temporally auto-correlated (1 h), this effect could be diluted (e.g., 72.4% and 71.4% for 15 min and 1 h, respectively). To the best of our knowledge, this represents the first time that this interaction has been tested, especially using such short fix intervals. Further research is needed to characterize better the effect of a ‘warm-start’ and antenna position on measurement error. Additionally, similar to previous studies performed in natural environments [29,35], the LE obtained with low-cost GPS data loggers was dependent on the habitat because the fixes performed in open habitats and near buildings were more accurate than those obtained inside buildings and under a closed coniferous cover (Table 5). However, even if the habitat did not include high-rise buildings, close proximity with human settlements significantly affected the satellite signals and GPS data-logger accuracy (e.g., 24.2% of fixes with LE < 10 m) despite presenting LE values that were similar to those previously reported in suburban environments (e.g., *m*LE = 30 m [14]).

The results from the controlled motion tests [33,35,36] and GPS devices mounted on free-ranging animals [24,28,30] generally indicated a reduction in GPS collar performance from stationary unit tests to motion tests (e.g., from 76% to 43% for stationary tests and collars deployed on the black bear *Ursus americanus* [34], respectively). We did not observe a similar reduction in accuracy, and our findings suggest that the FSR values between stationary and motion tests were similar. Our results were consistent with those reported by a recent study performed with low-cost GPS devices that reported higher FSR values with devices mounted on free-ranging otters compared to those tested in stationary conditions [15].

On average, our motion LE_RMS_ and FSR values were better than those from studies performed with a sled-like device that simulates domestic cat motion under various habitats [33] or devices attached to cars moving in open and forested areas [36] (Table 5). However, our FSR and fixes with LE < 10 m were similar to those reported in free-ranging animal returns [45] with a similar GPS data logger (I-gotU GT-120, Table 5). In addition, our LE_RMS_ and average LE values were comparable to those from motion tests performed for human epidemiology purposes [43,44] with the I-got-U GT-100 GPS data logger (Table 5). Similar to the results of the stationary unit tests, the LE increase was linked to a decrease in sky availability and traversal of forest habitat. We mimicked real data-collection conditions by testing the performance of mobile GPS data loggers carried by leashed dogs whose movements appeared to reduce the LE compared with stationary tests in forest habitat (LE_RMS_ = 40.9 m and 16.8 m in stationary and motion tests, respectively). However, the results should be interpreted cautiously because of our small sample size (two GPS data loggers on two dogs and four trips). Previous studies that performed stationary and motion tests concluded to a more realistic evaluation of performance for GPS collars based on the results derived from motion tests related to frequent changes in GPS position and direction or multipath crossings [33,35]. However, accurate motion tests are difficult to conduct because the accuracy of the reference position is reduced, the collar orientation is not controlled (i.e., low FSR associated with animal activity [61]) and various fix intervals are not used. In all of the motion tests conducted here, we ensured that the reference tracks were on the designed paths crossing habitats, although inherent error can be associated with the reference GPS. Additionally, the shortest fix interval was used to ensure a ‘hot-start’ of the reference GPS, the reference points were interpolated in time, and both units were synchronized.

## Conclusions

By evaluating the performance and accuracy of the GPS CatLog data logger near the ground and by incorporating the effects of antenna positions, fix intervals, habitats and movements, we adopted a conservative approach to determining the fix rate reduction and measurement error for medium-sized terrestrial mammals. Additionally, because we developed error estimates for habitats in rural environments, our results may be comparable to those of other research conducted in both natural and human-impacted flat landscapes.

Although the GPS devices were placed near the ground, our results emphasized that antenna movements that mimic medium-sized species behaviors, proximity to human settlements and canopy cover did not generate missing value biases. Thus, the performance in terms of FSR of the CatLog GPS data loggers appears to be efficient for tracking a wide range of terrestrial species, from urban-adapter to forest-specialized species. Additionally, while unsuccessful fix attempts may be the result of device failure (i.e., simple software malfunctioning, [9,15]), the impressive reduction in the FSR in human settlements can also be indicative of resting sites (i.e., failure caused by temporary blockage to satellites, [62]), and provide useful information on recursions and the importance of building use by certain terrestrial mammals. Indeed, blocks of unsuccessful fixes can represent time periods that a species spends in locations with poor satellite reception due to a dense forest canopy [37] or other physical cover (e.g., tree hollow for possums [45], subterranean dens for African leopards *Panthera pardus* [63], nesting habitats for European hedgehogs [16], etc.). Further research is needed to identify the type of failure responsible for consecutive unsuccessful fixes.

Our findings also illustrated that CatLog GPS data loggers often presented the same accuracy as layered habitat maps created by GIS software (50–70% of the LE values < 10 m, see also [45]) and were as accurate as large and lightweight built-in wildlife GPS units tested in both natural and anthropogenic landscapes. However, large LE values (LE_RMS_ > 40 m) can be associated with down antennas, short-fix intervals and sky obstructions (human settlements and forested areas). Compared with built-in wildlife GPS collars, CatLog GPS data loggers do not provide information on the number of available satellites for a given positional fix (i.e., fixes quality). These technological limitations suggest that when using CatLog GPS data loggers, the risk of acquiring fixes with reduced accuracy must be considered because a lower number of satellites may be available, which occurs when the GPS data logger antenna is downward with a 5-min fix interval or when the GPS data logger is in human settlements.

The influence of LE values depends on the total amount of movement and is associated with research focus and types of tracked species. For instance, studying migration dynamics (e.g., birds, buffalos and marine mammals moving from nesting to foraging habitats) can accommodate conclusive inferences with large LE values, and estimating home range sizes and shapes may require less accuracy. However, studying the fine-scale dynamics of movements of animals requires more precise measurements of the dimensions of habitat patches and movement patterns of the species of interest. For instance, studying the dynamics of cat movements within a household and in human settlements requires high accuracy that cannot be produced by CatLog data loggers. However, these data loggers appear to be suitable for studying medium-size terrestrial mammal fine-scale movements in open habitats, such as pastures and meadows. To accommodate research outcomes with inherent measurement errors, GPS tags can be associated with tri-axial accelerometer data loggers to identify location errors that could not be directly associated with animal movements.

## Supporting Information

S1 Fig Stationary unit tests. The stationary experimental arrangement conducted with 40 GPS CatLog data loggers (● = one GPS) (a) mounted on a plastic bottle, (b) arranged on a 1.5-m grid to test the effects of two collar orientations (-90° from horizontal, antenna down; +90° from horizontal, antenna up) and three fix intervals (5 min, program A; 15 min, program B; and 1 h, program C) in an open habitat and (c) arranged to test the influence of various habitats.
(TIF)

S2 Fig GPS data logger consistency. The cubic graphic illustrating the relationship of the median location error (m) according to the deployment time (h).
(TIF)

S1 Table Models explaining the location error (LE) of low-cost, lightweight GPS data loggers tested in stationary conditions (n = 24) with different antenna positions and fix intervals. (DOCX)

S2 Table Models explaining the location error (LE) of low-cost, lightweight GPS data loggers obtained during the controlled motion tests. (DOCX)

## References

[1] Cagnacci F, Boitani L, Powell RA, Boyce MS. Animal ecology meets GPS-based radiotelemetry: a perfect storm of opportunities and challenges. Philosophical Transactions of the Royal Society B: Biological Sciences 2010; 365: 2157–2162. 10.1098/rstb.2010.0107 20566493 PMC2894970

[2] Urbano F, Cagnacci F, Calenge C, Dettki H, Cameron A, Neteler M. Wildlife tracking data management: a new vision. Philosophical Transactions of the Royal Society B: Biological Sciences 2010; 365: 2177–2185. 10.1098/rstb.2010.0081 PMC2894960 20566495

[3] Coelho CM, de Melo LFB, Sábato MAL, Rizel DN, Young RJ. A note on the use of GPS collars to monitor wild maned wolves Chrysocyon brachyurus (Illiger 1815) (Mammalia, Canidae). Applied Animal Behaviour Science 2007; 105: 259–264. Available: 10.1016/j.applanim.2006.04.024.

[4] Frair JL, Nielsen SE, Merrill EH, Lele SR, Boyce MS, Munro RHM, et al Removing GPS collar bias in habitat selection studies. Journal of Applied Ecology 2004; 41: 201–212. 10.1111/j.0021-8901.2004.00902.x

[5] Soutullo A, Cadahia L, Urios V, Ferrer M, Negro JJ. Accuracy of Lightweight Satellite Telemetry: A Case Study in the Iberian Peninsula. The Journal of Wildlife Management 2007; 71: 1010–1015. 10.2193/2006-042

[6] Douglas-Hamilton I, Krink T, Vollrath F. Movements and corridors of African elephants in relation to protected areas. Naturwissenschaften 2005; 92: 158–163. 10.1007/s00114-004-0606-9 15770465

[7] Gurarie E, Andrews RD, Laidre KL. A novel method for identifying behavioural changes in animal movement data. Ecology Letters 2009; 12: 395–408. 10.1111/j.1461-0248.2009.01293.x 19379134

[8] Williams DM, Dechen Quinn A, Porter WF. Impact of Habitat-Specific GPS Positional Error on Detection of Movement Scales by First-Passage Time Analysis. PLoS ONE 2012; 7: e48439 10.1371/journal.pone.0048439 23144884 PMC3492345

[9] Brendel C, Helder R, Chevallier D, Zaytoon J, Georges J-Y, Handrich Y. Testing a global positioning system on free ranging badgers *Meles meles* . Mammal Notes, The Mammal Society, Southampton 2012: 1–5.

[10] Ferreira JP, Leitão I, Santos-Reis M, Revilla E. Human-related factors regulate the spatial ecology of domestic cats in sensitiveareas for conservation. PLoS ONE 2011; 6: e25970 10.1371/journal.pone.0025970 22043298 PMC3197152

[11] Recio MR, Mathieu R, Maloney R, Seddon PJ. First results of feral cats (*Felis catus*) monitored with GPS collars in New Zealand. New Zealand Journal of Ecology 2010; 34: 114–117.

[12] van Heezik Y, Smyth A, Adams A, Gordon J. Do domestic cats impose an unsustainable harvest on urban bird populations? Biological Conservation 2010; 143: 121–130.

[13] Horn JA, Mateus-Pinilla N, Warner RE, Heske EJ. Home range, habitat use, and activity patterns of free-roaming domestic cats. The Journal of Wildlife Management 2011; 75: 1177–1185. 10.1002/jwmg.145

[14] Adams AL, Dickinson KJM, Robertson BC, van Heezik Y. An Evaluation of the Accuracy and Performance of Lightweight GPS Collars in a Suburban Environment. PLoS ONE 2013; 8: e68496 10.1371/journal.pone.0068496 23874645 PMC3706378

[15] Quaglietta L, Martins BH, de Jongh A, Mira A, Boitani L. A Low-Cost GPS GSM/GPRS Telemetry System: Performance in Stationary Field Tests and Preliminary Data on Wild Otters (*Lutra lutra*). PLoS ONE 2012; 7: e29235 10.1371/journal.pone.0029235 22242163 PMC3252312

[16] Glasby L, Yarnell RW. Evaluation of the performance and accuracy of Global Positioning System bug transmitters deployed on a small mammal. European Journal of Wildlife Research 2013; 59: 915–919.

[17] Rose E, Nagel P, Haag-Wackernagel D. Spatio-temporal use of the urban habitat by feral pigeons (Columba livia). Behavioral Ecology and Sociobiology 2006; 60: 242–254. 10.1007/s00265-006-0162-8

[18] Cooke SJ, Hinch SG, Wikelski M, Andrews RD, Kuchel LJ, Wolcott TG, et al Biotelemetry: a mechanistic approach to ecology. Trends in Ecology & Evolution 2004; 19: 334–343. Available: 10.1016/j.tree.2004.04.003. 16701280

[19] Hebblewhite M, Haydon DT. Distinguishing technology from biology: a critical review of the use of GPS telemetry data in ecology. Philosophical Transactions of the Royal Society B: Biological Sciences 2010; 365: 2303–2312. 10.1098/rstb.2010.0087 20566506 PMC2894965

[20] Rodgers AR. Recent telemetry technology In: Millspaugh JJ, Marzluff JM, editors. Radio tracking and animal population. San Diego, CA: Academic Press; 2001 pp. 79–121.

[21] Hulbert IAR, French J. The accuracy of GPS for wildlife telemetry and habitat mapping. Journal of Applied Ecology 2001; 38: 869–878. 10.1046/j.1365-2664.2001.00624.x

[22] Frair JL, Fieberg J, Hebblewhite M, Cagnacci F, DeCesare NJ, Pedrotti L. Resolving issues of imprecise and habitat-biased locations in ecological analyses using GPS telemetry data. Philosophical Transactions of the Royal Society B: Biological Sciences 2010; 365: 2187–2200. 10.1098/rstb.2010.0084 PMC2894963 20566496

[23] D'Eon RG, Delparte D. Effects of radio-collar position and orientation on GPS radio-collar performance, and the implications of PDOP in data screening. Journal of Applied Ecology 2005; 42: 383–388. 10.1111/j.1365-2664.2005.01010.x

[24] Lewis JS, Rachlow JL, Garton EO, Vierling LA. Effects of habitat on GPS collar performance: using data screening to reduce location error. Journal of Applied Ecology 2007; 44: 663–671. 10.1111/j.1365-2664.2007.01286.x

[25] Webb SL, Dzialak MR, Mudd JP, Winstead JB. Developing spatially-explicit weighting factors to account for bias associated with missed GPS fixes in resource selection studies. Wildlife Biology 2013; 19: 257–273. 10.2981/12-038

[26] DeCesare NJ, Squires JR, Kolbe JA. Effect of forest canopy on GPS-based movement data. Wildlife Society Bulletin 2005; 33: 935–941. 10.2193/0091-7648(2005)33[935:eofcog]2.0.co;2

[27] Villepique JT, Bleich VC, Pierce BM, Stephenson TR, Botta RA, Bowyer RT. Evaluating GPS collar error: a critical evaluation of Televilt POSREC-Science (TM) collars and a method for screening location data. California Fish and Game 2008; 94: 155–168.

[28] Cain JW III, Krausman PR, Jansen BD, Morgart JR. Influence of topography and GPS fix interval on GPS collar performance. Wildlife Society Bulletin 2005; 33: 926–934. 10.2193/0091-7648(2005)33[926:iotagf]2.0.co;2

[29] Hansen MC, Riggs RA. Accuracy, Precision, and Observation Rates of Global Positioning System Telemetry Collars. The Journal of Wildlife Management 2008; 72: 518–526. 10.2193/2006-493

[30] Hebblewhite M, Percy M, Merrill EH. Are All Global Positioning System Collars Created Equal? Correcting Habitat-Induced Bias Using Three Brands in the Central Canadian Rockies. The Journal of Wildlife Management 2007; 71: 2026–2033. 10.2193/2006-238

[31] Bjørneraas K, Van Moorter B, Rolandsen CM, Herfindal I. Screening Global Positioning System Location Data for Errors Using Animal Movement Characteristics. The Journal of Wildlife Management 2010; 74: 1361–1366. 10.1111/j.1937-2817.2010.tb01258.x

[32] Humphries NE, Weimerskirch H, Sims DW. A new approach for objective identification of turns and steps in organism movement data relevant to random walk modelling. Methods in Ecology and Evolution 2013; 4: 930–938.

[33] Recio MR, Mathieu R, Denys P, Sirguey P, Seddon PJ. Lightweight GPS-Tags, One Giant Leap for Wildlife Tracking? An Assessment Approach. PLoS ONE 2011; 6: e28225 10.1371/journal.pone.0028225 22163286 PMC3233555

[34] Sager-Fradkin KA, Jenkins KJ, Hoffman RA, Happe PJ, Beecham JJ, Wright RG. Fix Success and Accuracy of Global Positioning System Collars in Old-Growth Temperate Coniferous Forests. Journal of Wildlife Management 2007; 71: 1298–1308. 10.2193/2006-367

[35] Cargnelutti B, Coulon A, Hewison AJM, Goulard M, Angibault J-M, Morellet N. Testing Global Positioning System Performance for Wildlife Monitoring Using Mobile Collars and Known Reference Points. The Journal of Wildlife Management 2007; 71: 1380–1387. 10.2193/2006-257

[36] Jiang Z, Sugita M, Kitahara M, Takatsuki S, Goto T, Yoshida Y. Effects of habitat feature, antenna position, movement, and fix interval on GPS radio collar performance in Mount Fuji, central Japan. Ecological Research 2008; 23: 581–588. 10.1007/s11284-007-0412-x

[37] Bourgoin G, Garel M, Dubray D, Maillard D, Gaillard J-M. What determines global positioning system fix success when monitoring free-ranging mouflon? European Journal of Wildlife Research 2009; 55: 603–613.

[38] Mattisson J, Andrén H, Persson J, Segerström P. Effects of Species Behavior on Global Positioning System Collar Fix Rates. The Journal of Wildlife Management 2010; 74: 557–563. 10.2193/2009-157

[39] Reid N, Harrison AT. Post-release GPS tracking of hand-reared Irish hare *Lepus timidus hibernicus* leverets, Slemish, Co. Antrim, Northern Ireland. Conservation Evidence 2010; 7: 32–38.

[40] Dean B, Freeman R, Kirk H, Leonard K, Phillips RA, Perrins CM, et al Behavioural mapping of a pelagic seabird: combining multiple sensors and a hidden Markov model reveals the distribution of at-sea behaviour. Journal of the Royal Society Interface 2012 10.1098/rsif.2012.0570 PMC3565783 23034356

[41] Thomas R, Baker P, Fellowes ME. Ranging characteristics of the domestic cat (Felis catus) in an urban environment. Urban Ecosystems 2014: 1–11. 10.1007/s11252-014-0360-5

[42] Parsons MB, Gillespie TR, Lonsdorf EV, Travis D, Lipende I, Gilagiza B, et al Global Positioning System Data-Loggers: A Tool to Quantify Fine-Scale Movement of Domestic Animals to Evaluate Potential for Zoonotic Transmission to an Endangered Wildlife Population. PLoS ONE 2014; 9: e110984 10.1371/journal.pone.0110984 25365070 PMC4217739

[43] Vazquez-Prokopec G, Stoddard S, Paz-Soldan V, Morrison A, Elder J, Kochel T, et al Usefulness of commercially available GPS data-loggers for tracking human movement and exposure to dengue virus. International Journal of Health Geographics 2009; 8: 68 10.1186/1476-072X-8-68 19948034 PMC2792221

[44] Stothard JR, Sousa-Figueiredo JC, Betson M, Seto EYW, Kabatereine NB. Investigating the spatial micro-epidemiology of diseases within a point-prevalence sample: a field applicable method for rapid mapping of households using low-cost GPS-dataloggers. Transactions of the Royal Society of Tropical Medicine and Hygiene 2011; 105: 500–506. 10.1016/j.trstmh.2011.05.007 21714979 PMC3183225

[45] Allan BM, Arnould JPY, Martin JK, Ritchie EG. A cost-effective and informative method of GPS tracking wildlife. Wildlife Research 2013; 40: 345–348. Available: 10.1071/WR13069.

[46] Wells AG, Wallin DO, Rice CG, Chang W-Y. GPS Bias Correction and Habitat Selection by Mountain Goats. Remote Sensing 2011; 3: 435–459.

[47] Belant JL. Effects of Antenna Orientation and Vegetation on Global Positioning System Telemetry Collar Performance. Northeastern Naturalist 2009; 16: 577–584. 10.1656/045.016.n407

[48] Dennis TE, Chen WC, Shah SF, Walker MM, Laube P, Forer P. Performance Characteristics of Small Global-Positioning-System Tracking Collars. Wildlife Biology in Practice 2010; 6: 14–31.

[49] Buerkert A, Schlecht E. Performance of three GPS collars to monitor goats’ grazing itineraries on mountain pastures. Computers and Electronics in Agriculture 2009; 65: 85–92. Available: 10.1016/j.compag.2008.07.010.

[50] Gompper ME. Top carnivores in the suburbs? Ecological and conservation issues raised by colonization of North-eastern North America by coyotes. Bioscience 2002; 52: 185–190. 10.1641/0006-3568(2002)052[0185:tcitse]2.0.co;2

[51] Gloor S, Bontadina F, Hegglin D, Deplazes P, Breitenmöser U. The rise of urban fox populations in Switzerland. Mammalian Biology 2001; 66: 155–164.

[52] Prange S, Gerht SD, Wiggers EP. Demographic factors contributing to high raccoon densities in urban landscapes. Journal of Wildlife Management 2004; 67: 324–333.

[53] Hoffmann IE, Milesi E, Pieta K, Dittami JP. Anthropogenic effects on the population ecology of European ground squirrels (*Spermophilus citellus*) at the periphery of their geographic range. Mammalian Biology 2003; 68: 205–213.

[54] Hubert P, Julliard R, Biagianti S, Poulle ML. Ecological factors driving the higher hedgehog (*Erinaceus europeaus*) density in an urban area compared to the adjacent rural area. Landscape and Urban Planning 2011; 103: 34–43.

[55] Sirguey P, Mathieu R, Arnaud Y. Subpixel monitoring of the seasonal snow cover with MODIS at 250 m spatial resolution in the Southern Alps of New Zealand: Methodology and accuracy assessment. Remote Sensing of Environment 2009; 113: 160–181. Available: 10.1016/j.rse.2008.09.008.

[56] Zuur A, Ieno EN, Walker N, Saveliev AA, Smith GM. Mixed effects models and extensions in ecology with R: Springer; 2009.

[57] Zuur AF, Ieno EN, Smith GM. Analysing ecological data: Springer New York; 2007.

[58] RCoreTeam (2014) R: A language and environment for statistical computing. R Foundation for Statistical Computing, Vienna, Austria. Available: http://www.R-project.org/.

[59] Zuur AF, Ieno EN, Elphick CS. A protocol for data exploration to avoid common statistical problems. Methods in Ecology and Evolution 2010; 1: 3–14. 10.1111/j.2041-210X.2009.00001.x

[60] Tomkiewicz SM, Fuller MR, Kie JG, Bates KK. Global positioning system and associated technologies in animal behaviour and ecological research. Philosophical Transactions of the Royal Society B: Biological Sciences 2010; 365: 2163–2176. 10.1098/rstb.2010.0090 PMC2894966 20566494

[61] D'Eon RG. Effects of stationary GPS fix-rate bias on habitat selection analyses. Journal of Wildlife Management 2003; 67: 858–863.

[62] Forin-Wiart M-A. Identification des facteurs de variation de la prédation exercée par les chats domestiques (*Felis silvestris catus*) en milieu rural. Ph.D. Thesis, Université de Reims Champagne-Ardenne. 2014. Available: http://www.theses.fr/2014REIMS032/abes.

[63] Swanepoel LH, Dalerum F, Van Hoven W. Factors affecting location failure of GPS collars fitted to African leopards (Panthera pardus). South African Journal of Wildlife Research 2010; 40: 10–15.

