# Supplementary figures and images for: Performance and Accuracy of Lightweight and Low-Cost GPS Data Loggers According to Antenna Positions, Fix Intervals, Habitats and Animal Movements
Source: PLoS One. 2015 Jun 18;10(6):e0129271. doi: 10.1371/journal.pone.0129271 (PMC4472960; doi:10.1371/journal.pone.0129271)

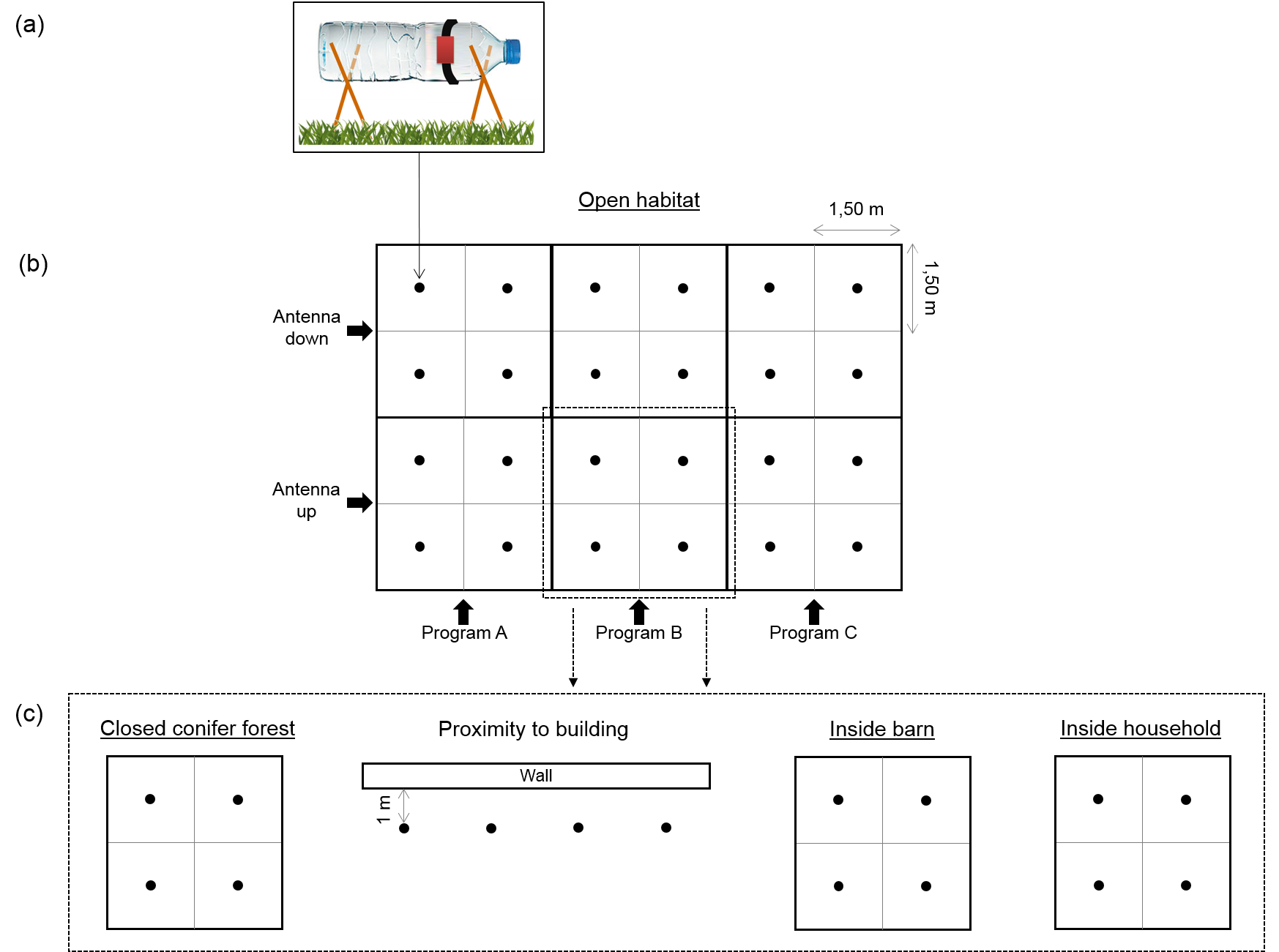

Supplement: S1 Fig — The stationary experimental arrangement conducted with 40 GPS CatLog data loggers (● = one GPS) (a) mounted on a plastic bottle, (b) arranged on a 1.5-m grid to test the effects of two collar orientations (-90° from horizontal, antenna down; +90° from horizontal, antenna up) and three fix intervals (5 min, program A; 15 min, program B; and 1 h, program C) in an open habitat and (c) arranged to test the influence of various habitats. (TIF) [file pone.0129271.s001.tif]

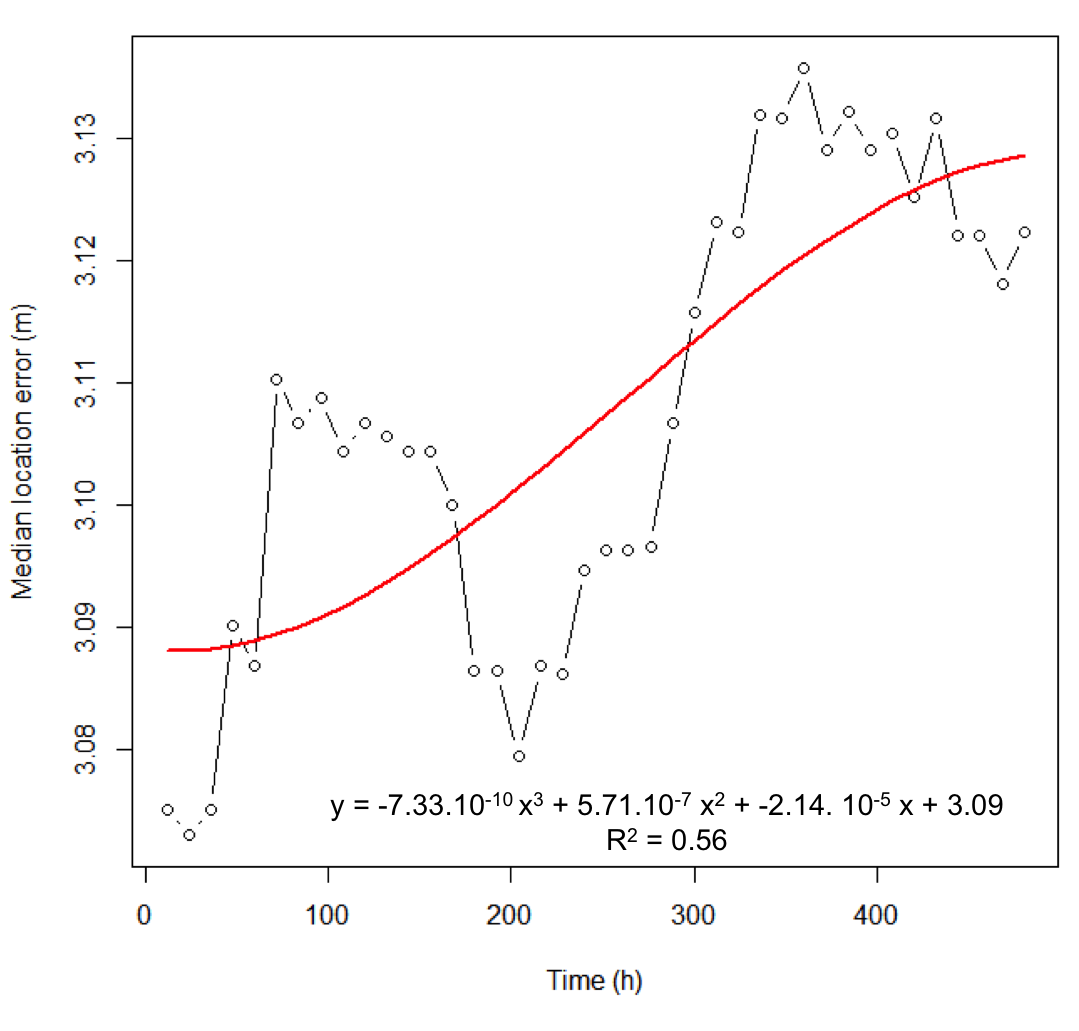

Supplement: S2 Fig — The cubic graphic illustrating the relationship of the median location error (m) according to the deployment time (h). (TIF) [file pone.0129271.s002.tif]
