# Supplementary material for: Performance and Accuracy of Lightweight and Low-Cost GPS Data Loggers According to Antenna Positions, Fix Intervals, Habitats and Animal Movements
Source: PLoS One. 2015 Jun 18;10(6):e0129271. doi: 10.1371/journal.pone.0129271 (PMC4472960; doi:10.1371/journal.pone.0129271)
Supplement: S1 Table — (DOCX) [file pone.0129271.s003.docx]

**Table S1. Models explaining the location error (LE) of low-cost, lightweight GPS data loggers tested in stationary conditions (n = 24) with different antenna positions and fix intervals.**

| **Rank** | **Model description** | **K** | **LL** | **AIC** | **∆AIC** | ***ω_i_*** |
| --- | --- | --- | --- | --- | --- | --- |
| 1 | fAntenna + fFix + fAntenna: fFix | 8 | - 4477.8 | 10013.4 | 0.00 | 0.96 |
| 2 | fAntenna + fFix | 6 | - 5004.0 | 10020.0 | 6.59 | 0.04 |
| 3 | fAntenna | 4 | - 5009.6 | 10027.1 | 13.73 | 0.00 |
| 4 | Null | 3 | - 5032.5 | 10071.1 | 57.68 | 0.00 |
| 5 | fFix | 5 | - 5031.8 | 10073.7 | 60.34 | 0.00 |
