# Supplementary material for: Performance and Accuracy of Lightweight and Low-Cost GPS Data Loggers According to Antenna Positions, Fix Intervals, Habitats and Animal Movements
Source: PLoS One. 2015 Jun 18;10(6):e0129271. doi: 10.1371/journal.pone.0129271 (PMC4472960; doi:10.1371/journal.pone.0129271)
Supplement: S2 Table — (DOCX) [file pone.0129271.s004.docx]

**Table S2. Models explaining the location error (LE) of low-cost, lightweight GPS data loggers obtained during the controlled motion tests.**

| **Rank** | **Model description** | **K** | **LL** | **AICc** | **∆AICc** | ***ω_i_*** |
| --- | --- | --- | --- | --- | --- | --- |
| 1 | fHabCross | 5 | - 556.8 | 1123.6 | 0.00 | 0.30 |
| 2 | fHabCross + fUnit + fPath | 8 | - 554.0 | 1123.9 | 0.33 | 0.25 |
| 3 | fHabCross + fPath | 8 | - 554.0 | 1123.9 | 0.33 | 0.25 |
| 4 | fHabCross + fUnit | 6 | - 556.2 | 1124.4 | 0.85 | 0.19 |
| 5 | Vd | 3 | - 587.9 | 1181.8 | 58.26 | 0.00 |
| 6 | Vd + fUnit | 4 | - 587.5 | 1183.1 | 59.51 | 0.00 |
| 7 | Vd + fUnit + fPath | 6 | - 586.2 | 1184.4 | 60.78 | 0.00 |
| 8 | Vd + fPath | 6 | - 586.2 | 1184.4 | 60.78 | 0.00 |
| 9 | Null | 2 | - 601.0 | 1205.9 | 82.29 | 0.00 |
| 10 | fUnit | 3 | - 600.6 | 1207.2 | 83.60 | 0.00 |
| 11 | fUnit + fPath | 5 | - 600.4 | 1210.9 | 87.28 | 0.00 |
| 12 | fPath | 5 | - 600.4 | 1210.9 | 87.28 | 0.00 |
